# A randomised controlled trial of supplemental oxygen versus medical air during exercise training in people with chronic obstructive pulmonary disease: supplemental oxygen in pulmonary rehabilitation trial (SuppORT) (Protocol)

**DOI:** 10.1186/s12890-016-0186-4

**Published:** 2016-02-04

**Authors:** Jennifer A Alison, Zoe J McKeough, Sue C Jenkins, Anne E Holland, Kylie Hill, Norman R Morris, Regina WM Leung, Kathleen A Williamson, Lissa M Spencer, Catherine J Hill, Annemarie L Lee, Helen Seale, Nola Cecins, Christine F McDonald

**Affiliations:** Discipline of Physiotherapy, Faculty of Health Sciences, The University of Sydney, Sydney, Australia; Department of Physiotherapy, Royal Prince Alfred Hospital, Sydney, Australia; Institute for Respiratory Health, Perth, Australia; Department of Physiotherapy, Sir Charles Gairdner Hospital, Perth, Australia; School of Physiotherapy and Exercise Science, Faculty of Health Sciences, Curtin University, Perth, Australia; Discipline of Physiotherapy, School of Allied Health, La Trobe University, Melbourne, Australia; Department of Physiotherapy, Alfred Health, Melbourne, Australia; Institute for Breathing and Sleep, Melbourne, Australia; Menzies Health Institute and School of Allied Health Sciences, Griffith University, Brisbane, Australia; The Queensland Lung Transplant Service, The Prince Charles Hospital, Brisbane, Australia; Department of Respiratory and Sleep Medicine, Concord Repatriation General Hospital, Sydney, Australia; Department of Physiotherapy, Austin Health, Melbourne, Australia; Department of Respiratory and Sleep Medicine, Austin Health, Melbourne, Australia; Department of Medicine, The University of Melbourne, Melbourne, Australia

**Keywords:** Chronic obstructive pulmonary disease (COPD), Exercise training, Oxygen desaturation, Supplemental oxygen

## Abstract

**Background:**

Oxygen desaturation during exercise is common in people with chronic obstructive pulmonary disease (COPD). The aim of the study is to determine, in people with COPD who desaturate during exercise, whether supplemental oxygen during an eight-week exercise training program is more effective than medical air (sham intervention) in improving exercise capacity and health-related quality of life both at the completion of training and at six-month follow up.

**Methods/Design:**

This is a multi-centre randomised controlled trial with concealed allocation, blinding of participants, exercise trainers and assessors, and intention-to-treat analysis. 110 people with chronic obstructive pulmonary disease who demonstrate oxygen desaturation lower than 90 % during the six-minute walk test will be recruited from pulmonary rehabilitation programs in seven teaching hospitals in Australia. People with chronic obstructive pulmonary disease on long term oxygen therapy will be excluded. After confirmation of eligibility and baseline assessment, participants will be randomised to receive either supplemental oxygen or medical air during an eight-week supervised treadmill and cycle exercise training program, three times per week for eight weeks, in hospital outpatient settings. Primary outcome measures will be endurance walking capacity assessed by the endurance shuttle walk test and health-related quality of life assessed by the Chronic Respiratory Disease Questionnaire. Secondary outcomes will include peak walking capacity measured by the incremental shuttle walk test, dyspnoea via the Dyspnoea-12 questionnaire and physical activity levels measured over seven days using an activity monitor. All outcomes will be measured at baseline, completion of training and at six-month follow up.

**Discussion:**

Exercise training is an essential component of pulmonary rehabilitation for people with COPD. This study will determine whether supplemental oxygen during exercise training is more effective than medical air in improving exercise capacity and health-related quality of life in people with COPD who desaturate during exercise.

**Trial registration:**

Australian New Zealand Clinical Trials Registry ACTRN12612000395831, 5th Jan,2012

## Background

Pulmonary rehabilitation is an important component of the management of people with chronic obstructive pulmonary disease (COPD) with strong evidence of efficacy [1]. Clinically significant improvements in exercise capacity, symptoms of breathlessness, fatigue and health-related quality of life (HRQoL) are consistently documented in randomised controlled trials of pulmonary rehabilitation [1]. The magnitude of benefit in exercise capacity may be dependent on the intensity of exercise training. In people with COPD, there is some evidence to suggest that training at higher intensities confers greater physiological changes that promote improved exercise capacity [2] and therefore high intensity exercise training is recommended in pulmonary rehabilitation programs [3].

Exercise-induced oxygen desaturation is common among people with COPD, with 47 % of those referred to pulmonary rehabilitation demonstrating a decrease in oxygen saturation to less than 90 % during a six-minute walk test (6MWT) [4]. Exercise-induced desaturation may compromise the intensity of training since those who desaturate often cannot tolerate high intensity exercise [3] and healthcare professionals may strive to minimise exercise-induced desaturation by decreasing training intensity and/or imposing mandatory rests. This reduction in exercise intensity is likely to limit the effectiveness of training [2]. Provision of supplemental oxygen reduces minute ventilation at equivalent work rates in people with COPD and delays the onset of dynamic hyperinflation and the associated dyspnoea [5, 6], thus augmenting exercise capacity in people with moderate to severe COPD [7]. Therefore, supplemental oxygen may enable higher exercise intensity during an exercise training program [8].

For these reasons, supplemental oxygen is often used in pulmonary rehabilitation for people with COPD, especially in those patients who desaturate during exercise [4]. However, evidence to support the use of supplemental oxygen during pulmonary rehabilitation in this patient group is lacking. Since the provision of supplemental oxygen during pulmonary rehabilitation requires trained staff, increases program costs and restricts the locations in which training can be delivered, strong evidence to support or refute the use of supplemental oxygen for those people with COPD who desaturate during exercise is required.

The primary aim of the study is to determine, in people with COPD who desaturate during exercise, whether supplemental oxygen during exercise training is more effective than medical air (sham intervention) in improving endurance exercise capacity and HRQoL, at the completion of exercise training and at six-month follow-up.

The secondary aim is to determine, in people with COPD who desaturate during exercise, whether supplemental oxygen during exercise training is more effective than medical air in improving walking capacity, reducing dyspnoea and increasing levels of daily physical activity, at the completion of pulmonary rehabilitation and at six-month follow-up.

## Methods

### Design

This study will be a prospective, multi-centre, randomised controlled trial with concealed allocation, blinding of participants, therapists and assessors and with intention-to-treat analysis.

### Participants

People will be eligible for inclusion if they have: a diagnosis of COPD (post-bronchodilator forced expiratory volume in one second (FEV_1_) / forced vital capacity (FVC) ratio of <0.7; a greater than 10 pack-year smoking history; medically stable (at least four weeks post an exacerbation); and evidence of oxygen desaturation to less than 90 % during the 6MWT performed on room air. Participants with co-existing cardiac conditions, such as controlled atrial fibrillation and controlled heart failure, will be included to ensure that the sample population is representative and reflective of patients currently referred to pulmonary rehabilitation programs. Informed written consent will be obtained from all participants and the study has been approved by the Ethics Committees of all participating sites (Human Research Ethics Committee of Sydney Local Health District, South Western Sydney Local Health District, Austin Health, The Prince Charles Hospital and Curtin University). The trial is registered with Australian New Zealand Trial Registry: ACTRN12612000395831.

People will be excluded if they are receiving long-term oxygen therapy, have a resting partial pressure of arterial oxygen (PaO_2_) on room air of < 55 mmHg or a partial pressure of arterial carbon dioxide (PaCO_2_) of > 50 mmHg, have participated in any supervised exercise training in the last 12 months or if they have co-morbidities such as severe cardiovascular, neurological or musculoskeletal conditions that are likely to adversely affect performance during assessments or exercise training.

**Fig. 1:**
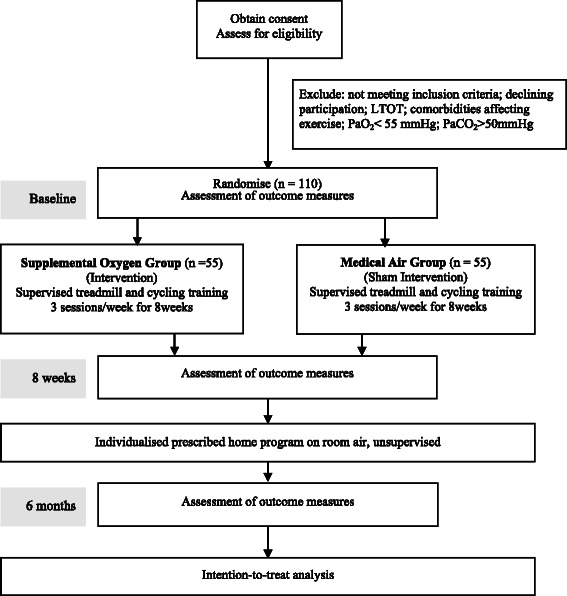
Flow of participants through the trial. LTOT = long term oxygen therapy

The flow of participants through the trial will reflect the recommendations from the Consolidated Standards of Reporting Trials (CONSORT) guidelines [9] (Fig. 1). Participants will receive written and verbal information explaining the study and written consent will be obtained from each participant. Ethics approval to conduct the study has been obtained from the Ethics Committees of the seven participating sites.

### Recruitment

Participants will be recruited from referrals to pulmonary rehabilitation programs at the participating sites (Royal Prince Alfred Hospital, Concord Repatriation General Hospital, Liverpool Hospital, Sydney; Austin Hospital and The Alfred Hospital, Melbourne; The Prince Charles Hospital, Queensland; Sir Charles Gairdner Hospital, Perth). Prior to randomisation participants will undergo screening tests to ensure eligibility.

### Screening tests

Participants will perform two 6MWTs while breathing room air to ensure that they meet the study criteria related to oxygen desaturation on exertion. Oxygen saturation (SpO_2_) will be monitored continuously through the 6MWTs via a finger probe attached to a portable saturation monitor (RAD-5v Masimo Corp, Irvine, CA, USA). The nadir SpO_2_ from the better 6MWT (i.e. the greatest distance walked) will be used as the measure of oxygen desaturation.

Additional measures undertaken prior to randomisation (i.e. baseline) will comprise spirometry, resting lung volumes, single breath diffusing capacity for carbon monoxide (D_L_CO) and PaO_2_ and PaCO_2_, whilst breathing room air [10–12].

### Randomisation

Participants who meet the study inclusion criteria and complete the baseline assessments will be randomly allocated into one of the two groups: Oxygen Group or Air Group. Equal numbers of participants will be randomised to each group. Sequence generation will be determined using a computerised random number generator with stratification for study site, six-minute walk distance (6WMD) (≤350 metres vs >350 metres) and level of nadir SpO_2_ from the 6MWT (nadir SpO_2_ between 89-86 % vs < 86 %). Allocation concealment will be achieved by the use of a central telephone randomisation system coordinated through the National Health and Medical Research Council Clinical Trials Centre at The University of Sydney. This will ensure that the researchers are blinded to the sequence generation.

### Blinding

Participants, exercise trainers and assessors will be blinded as to whether the participants are receiving oxygen or medical air. Oxygen concentrators (Air Liquide, Healthcare Pty Ltd, Australia) will be used for the delivery of supplemental oxygen to the Oxygen Group. Oxygen concentrators modified by Air Liquide with Therapeutic Goods Administration (TGA) approval to deliver medical air only, will be used for the Air Group. The concentrators for the Oxygen and Air Groups will be identical and coded by an independent body (Air Liquide, Healthcare Pty Ltd, Australia). The code to distinguish which intervention a participant is receiving will only be available to the randomisation centre.

### Experimental intervention

Both the Oxygen and Air Groups will participate in the same supervised exercise training program. The Oxygen Group will receive intranasal oxygen at 5 L/min from an oxygen concentrator during this exercise training program. The Air Group will receive intranasal air at 5 L/min from an oxygen concentrator modified to deliver air during this exercise training program. The fixed flow rate of 5 L/min will ensure adequate delivery of gas through the concentrators for both groups.

### Exercise training

Exercise training will consist of supervised treadmill walking and stationary cycling, performed three times per week for eight weeks. The training period will be extended to 10 weeks if training is interrupted by the inability to attend due to illness with the aim of completing a minimum of 20 sessions. The initial duration of exercise training will be 30 minutes (20 minutes treadmill walking and 10 minutes stationary cycling) and will be progressed up to a total of 40 minutes (20 minutes treadmill walking and 20 minutes stationary cycling) by week three.

The initial exercise intensity for each mode of training will be standardised across the Oxygen and Air Group. For the treadmill walking, the initial intensity will be set at 80 % of the participant’s average speed achieved during the 6MWT (based on the best 6MWT performed at the screening assessment). For stationary cycling, the initial intensity will be set at 60 % of the peak work rate estimated using the distance walked in the best 6MWT [13].

#### Progression of training

Participants will score dyspnoea and rate of perceived exertion (RPE) after each treadmill and cycle training session using the modified dyspnoea and RPE 0–10 scales [14, 15]. These scores will be used to guide the progression of both treadmill and cycle training, with the aim to keep either dyspnoea or RPE at a ‘moderate’ to ‘somewhat severe’ level (i.e. a score of 3 to 4).

To progress treadmill training, the walking speed will be increased once a participant completes three sessions at the prescribed speed. If the initial walking speed is < 3 km/hr, the speed will be increased by 0.25 km/hr. If the initial walking speed is >3 km/hr the speed will be increased by 0.5 km/hr. If the participant reports both dyspnoea and RPE scores < 3 after a treadmill training session, the speed for the next training session will be increased as tolerated. Once the participant has reached a walking speed of 5 km/hr, the speed will be reduced to 4.5 km/hr and a gradient of 1 to 2 % will be added. The gradient will be increased by 1 to 2 % after every three training sessions if symptoms of dyspnoea and RPE allow (i.e. dyspnoea and RPE scores < 3). If the participant experiences difficulty achieving a walking speed of 5 km/hr (e.g. unable to walk fast due to short stride length) and reports dyspnoea or RPE scores < 3, the gradient will be introduced earlier to ensure the exercise intensity is appropriate.

Cycle training will be progressed after six sessions if the participant reports dyspnoea or RPE scores < 3.The progression will be an increase in duration of cycle training by five minutes, to a maximum of 20 minutes. Once the participant is able to successfully perform three sessions of cycle training for 20 minutes and if dyspnoea and RPE scores are < 3, the work rate will be increased by 5 watts. Thereafter, for the remainder of the training program, the intensity of the cycle based training will be increased in 5 watt increments, once a participant completes three sessions at the prescribed intensity with dyspnoea and RPE scores <3.

#### Safety during the exercise training sessions

While oxygen desaturation during exercise has been shown to be common in people with COPD, a recent study showed that oxygen desaturation below 80 % during a 6MWT only occurred in 26 out of 572 participants (i.e. 4.5 %) [16]. In the current study, it is anticipated that an even smaller proportion will desaturate below 80 % during exercise training as the intensity of exercise will be less than the peak achieved during the 6MWT. In addition, half the participants in this study will be allocated to the Oxygen Group and will receive oxygen during their exercise training sessions, thereby ameliorating any desaturation. For this reason, the decision has been made that the trainers will not monitor SpO_2_ during the training program. This will ensure that they stay blinded to group allocation. Nevertheless, to ensure participant safety, SpO_2_ will be monitored and recorded throughout the training program by a clinician who is not involved in exercise prescription or progression. A standardised pulse oximeter (RAD-5v Masimo Corp, Irvine, CA, USA) will be used at all sites. The pulse oximeter will be hidden from the participant and trainer in order to maintain blinding. This clinician will also be blinded to group allocation and will not reveal the changes in SpO_2_ to the trainer unless SpO_2_ falls below 80 % [17], at which point the participant will be asked to stop exercising and rest. The participant will be instructed to recommence exercising when SpO_2_ returns to 88 %. The clinician will monitor the initial exercise training session and, thereafter, will monitor an entire training session once a week for the remainder of the training program.

### Participant education

At the end of the exercise training program an education booklet [18] will be provided and an individualised home maintenance exercise program will be given to all participants. All participants will be encouraged to complete the home exercise program for six months after which they will return for reassessment. No supplemental oxygen will be provided for the home program.

### Outcome measures

The primary outcomes will be endurance exercise capacity measured by the endurance shuttle walk test (ESWT) [19] and HRQoL measured by the Chronic Respiratory Disease Questionnaire (CRQ) [20]. The secondary outcomes will be peak exercise capacity measured by the incremental shuttle walk test (ISWT) [21], level of dyspnoea using the Dyspnoea-12 Questionnaire [22] and physical activity levels measured by a multi-sensor activity monitor (SenseWear MF, BodyMedia, Pittsburgh USA) worn for seven days.

An assessor who is blinded to group allocation will carry out all outcome measures at baseline, at the completion of exercise training and six months following completion of the exercise training program. All the exercise outcome measures at each time point, will be performed with the participant breathing room air.

### Primary outcomes

Endurance exercise capacity: Endurance exercise capacity will be measured by the ESWT, performed according to a published protocols [19]. The ESWT is an externally paced constant work rate test which is performed at a walking speed equivalent to 85 % of the participant’s peak speed achieved on the incremental shuttle walk test (ISWT) [23]. Participants will be asked to walk for as long as possible, back and forth along a 10 metre shuttle course, in time to a pre-recorded auditory signal. Dyspnoea and RPE will be recorded each minute during the test and at the beginning and end of the test using the modified Borg 0–10 Scale [14, 15]. Heart rate and SpO_2_ will be monitored continuously using a portable saturation monitor (RAD-5v Masimo Corp, Irvine, CA, USA) and recorded each minute. At the end of the test, each participant will be asked to identify the main reason for stopping the test.

The ESWT will be performed twice at all assessment time points. Participants will rest for at least 30 minutes between tests or until SpO_2_, dyspnoea and heart rate have returned to resting levels. Due to the ceiling effect of 20 minutes in the ESWT [19], the ESWT protocol will be modified so that if the participant completes more than 10 minutes in the first baseline ESWT, the test will be stopped and repeated at a higher level. The goal is to have each participant's baseline ESWT time between five and 10 minutes. This will allow the test to demonstrate improvement without reaching the 20 minute test termination at assessments after training. The walking speed for repeat testing of the ESWT after exercise training and at six-month follow-up will remain the same as that of the participant’s highest level baseline test. The greater time walked in the ESWT at each assessment time point will be recorded for analysis. The minimal important difference for the ESWT following ground walking training in people with COPD is 156 seconds [24].

HRQoL: The interviewer-administered version of the CRQ [20] will be used to measure disease-specific HRQoL. The questionnaire consists of 20 questions which are grouped into one of four domains: dyspnoea (5 questions), fatigue (4 questions), emotional functioning (7 questions) and mastery (4 questions). Each question is scored from one to seven, with a higher score indicating a better health status. A total score, as well as individual domain scores, will be calculated. The CRQ has been shown to be a reliable and valid tool for measuring HRQoL in people with chronic respiratory disease [20]. A change of 0.5 in the mean score per domain (calculated by dividing the overall score by the number of questions) has been shown to be associated with a minimal important difference in health status [25]. This means that a minimal important difference would be 2.5 for dyspnoea, 2 for fatigue, 3.5 for emotional function, 2 for mastery, and 10 for the total CRQ score.

### Secondary outcomes

Peak exercise capacity will be measured using the ISWT which is an incremental, externally paced, symptom-limited test with walking speed increasing each minute [26]. The participant will be asked to walk back and forth along the 10 metre shuttle course in time with the pre-recorded auditory signals. There are 12 one-minute levels, with each level requiring the participant to perform successively more shuttles in each minute. Dyspnoea and RPE will be recorded at the beginning and end of the test and each minute during the test using the modified Borg 0–10 Scale [14, 15]. Heart rate and SpO_2_ will be monitored continuously using a portable saturation monitor (RAD-5v Masimo Corp, Irvine, CA, USA) and recorded each minute. The test is terminated when the participant chooses to stop or when, on two consecutive auditory signals, the participant is more than one metre from the closest cone [26]. At the end of the test, participants will be asked to identify the main reason for stopping.

The ISWT will be performed twice at baseline and at subsequent assessments to account for a learning effect [26] and the greater distance recorded for analysis. Participants will rest for at least 30 minutes between tests or until SpO_2_, dyspnoea and heart rate have returned to resting levels. The minimal important difference for the ISWT for pulmonary rehabilitation is 47.5 metres (95 % confidence intervals 38.6 to 56.5 metres) [27].

Dyspnoea will be measured by the Dyspnoea-12 questionnaire [22]. The Dyspnoea-12 questionnaire consists of 12 descriptor items on a scale of none (zero point), mild (one point), moderate (two points), or severe (three points). It provides an overall score for severity of dyspnoea that incorporates seven items pertaining to the quality of the sensation of dyspnoea and five items pertaining to the emotional response to the sensation of dyspnoea. The time reference period for “these days” captures the current level of dyspnoea experienced by the participants as opposed to specifically on the day of the test or in response to a specific activity. The total score for the Dyspnoea-12 questionnaire ranges from 0 to 36, with higher scores corresponding to greater severity of dyspnoea.

Physical activity will be measured by a multi-sensor activity monitor (SenseWear MF, BodyMedia, Pittsburgh, USA). This device provides information on activity intensity via energy expenditure, duration of levels of energy expenditure and step count. The activity monitor will be worn continuously over a seven-day period at baseline, at the completion of exercise training and at the six-month follow-up assessment. Minimum wear time will be set at three days for at least 20 hours per day. This device is well tolerated in people with COPD [28] and has been shown to provide a reliable and valid measure of physical activity in people with COPD [29, 30].

### Additional measures

At each assessment time point, spirometry (EasyOne handheld Spirometer, ndd Medical Technologies, Chelmsford, MA) will be performed to determine whether the participant’s lung function remains stable during the study period.

### Data analysis

For all outcome measures the primary analyses will be via intention-to-treat and will be conducted by a statistician who has not been involved in the study and is unaware of group allocation. Data will be analysed using linear mixed models or analysis of covariance (ANCOVA) if there are between group differences at baseline. Uncertainty regarding the size of the mean between group differences will be quantified with 95 % confidence intervals.

### Sample size

A total of 110 participants (55 per group) will be recruited to ensure that 88 participants complete the study, allowing for a 20 % loss to follow-up. Regarding primary outcomes, 88 participants will be sufficient to provide 80 % power to detect as significant, at the (two-sided) 5 % level, a minimum 156 second difference [24] in the mean ESWT time between the Oxygen Group and the Air Group. This assumes a standard deviation of 250 seconds for the ESWT, as has been previously reported when comparing two modes of exercise training in COPD [31]. For the HRQoL outcome, 88 participants will be sufficient to provide 80 % power to detect as significant, at the (two-sided) 5 % level, a minimum 10 point difference [32] in the mean CRQ total score between the groups, assuming a standard deviation (SD) of 17 points [33].

Regarding secondary outcomes, 82 participants will be sufficient to provide 80 % power to detect as significant, at the (two-sided) 5 % level, a minimum of 2.5 point difference (a change of 0.5 point per question for a total of five questions in the dyspnoea domain) between groups in the mean score of the dyspnoea domain of the CRQ, assuming a SD of 4.0 [34]. For the physical activity outcome, 82 participants will be sufficient to provide 80 % power to detect as significant, at the (two-sided) 5 % level, a minimum of 1845 step difference in the mean steps per day between the groups, assuming a SD of 2968 steps for people with moderate to severe COPD [35].

## Discussion

The primary aim of this study is to determine if supplemental oxygen is more effective than medical air (sham intervention) during exercise training in improving endurance exercise capacity and HRQoL for people with COPD who desaturate during exercise. We hypothesise that: i) exercise training with supplemental oxygen will increase endurance exercise capacity and HRQoL more than exercise training with medical air; and ii) the difference in exercise capacity between groups will be maintained at a follow-up assessment undertaken six months following completion of the training period.

Evidence to support the use of supplemental oxygen during exercise training in people with COPD who desaturate during exercise is lacking. Three previous randomised controlled trials have evaluated the effects of supplemental oxygen during a program of supervised exercise training in people with COPD who experience oxygen desaturation on exertion and failed to show between group differences [36–38]. These trials had design limitations with lack of participant and/or investigator blinding to group allocation and small sample sizes (≤12 per group). Furthermore, as the studies did not report the progression of training intensity it is not clear whether the participants in the intervention groups were able to achieve a higher training intensity using supplemental oxygen.

In contrast with these earlier studies, a small study by Emtner et al. [8] used a double-blind placebo controlled randomised study design and showed that the group that received supplemental oxygen (*n* = 14) achieved higher training intensities and had significantly greater exercise endurance time compared with the group that trained using compressed air (*n* = 15). However, this study recruited people with moderate to severe COPD, who did not desaturate during exercise, therefore, the results cannot be applied to those who would typically be considered for supplemental oxygen during pulmonary rehabilitation programs. In addition, the study used cycle-based training exclusively, whereas most pulmonary rehabilitation programs in Australia [39-43] and other countries [44, 45] use both walking and cycle-based exercise. This is an important difference as the physiological responses to cycle and walking-based exercise differs significantly in people with COPD [46, 47]. Specifically, people with COPD demonstrate more profound oxygen desaturation during walking-based exercise compared to cycle-based exercise [47, 48] and therefore the effect of supplemental oxygen may differ between these two exercise modalities. Since the study by Emtner et al. [8] was based exclusively on both exercise training and testing using a cycle ergometer it remains unclear if supplemental oxygen confers benefits in terms of walking-based exercise capacity in those who desaturate during walking exercise. Walking is a common activity of daily living and thus gains in walking capacity are more likely to be important to people with COPD.

Oxygen desaturation < 90 % during exercise affects 47 % of people with COPD referred to pulmonary rehabilitation programs in Australia [16, 48]. Previous work has shown that those who desaturate during walking-based exercise gain improvement in exercise capacity, measured as six-minute walk distance (6MWD), with acute administration of supplemental oxygen [49]. Specifically, in those who desaturated ≥ 5 % to < 90 % during a 6MWT, supplemental oxygen increased 6MWD (i.e. an increase from 391 to 450 metres; p < 0.02) [49]. In contrast, no change in 6MWD was observed in response to supplemental oxygen in those who did not meet these criteria for desaturation [49]. The capacity of supplemental oxygen to confer gains in 6MWD only in those who desaturate may relate to its amelioration of the adverse pulmonary haemodynamics associated with exercise-induced hypoxaemia [50]. If supplemental oxygen during exercise training in people who desaturate is shown to be an effective intervention to improve exercise capacity when exercising without oxygen as well as to improve HRQoL, it has the capacity to enhance the outcomes of exercise training for nearly half of all people with COPD.

This proposed study will be the first multi-centre, double-blind randomised trial investigating the role of supplemental oxygen in optimising training intensity and maximising the benefits gained from pulmonary rehabilitation in people with COPD who experience oxygen desaturation during walking. The recruitment of participants from pulmonary rehabilitation programs across Australia will ensure that the study is sufficiently powered to detect any between group differences. In addition, the randomised allocation of participants and the blinding of the participants, exercise trainers and assessors will limit bias and contribute to the high quality assessment of clinical outcomes.

## Abbreviations

6MWT: Six-minute walk test
6MWD: Six-minute walk distance
ANCOVA: Analysis of covariance
CONSORT: Consolidated standards of reporting trials
COPD: Chronic obstructive pulmonary disease
CRQ: Chronic respiratory disease questionnaire
D_L_CO: Diffusion capacity of the lung for carbon monoxide
ESWT: Endurance shuttle walk test
FEV_1_: Forced expiratory volume in one second
FVC: Forced vital capacity
HRQoL: Health-related quality of life
Hr: Hour
ISWT: Incremental shuttle walk test
Km: Kilometres
L: Litres
min: Minute
PaCO_2_: Partial pressure of arterial carbon dioxide
PaO_2_: Partial pressure of arterial oxygen
RPE: Rate of perceived exertion
SpO_2_: Oxygen saturation
